# Comparison of perventricular and percutaneous ultrasound-guided device closure of perimembranous ventricular septal defects

**DOI:** 10.3389/fcvm.2023.1281860

**Published:** 2023-11-06

**Authors:** Liu Liu Huang, Mai Chen, De Cai Zeng, Chun Xiao Su, Chun Lan Jiang, Bao Shi Zheng, Ji Wu, Shi Kang Li

**Affiliations:** ^1^ Department of Cardiothoracic Surgery, The First Affiliated Hospital of Guangxi Medical University, Nanning, China; ^2^ Department of Ultrasound, The First Affiliated Hospital of Guangxi Medical University, Nanning, China

**Keywords:** congenital heart defect, ventricular septal defect (perimembranous type), percutaneous (catheter-based) treatment, perventricular device closure, echocardiography guidance

## Abstract

**Background:**

Ultrasound-guided percutaneous device closure of perimembranous ventricular septal defects (PmVSD) is a minimally invasive recent treatment approach. Perventricular PmVSD device closure is an emerging radiation-free intervention, yet it comes with certain limitations. No studies compared both of these treatment approaches.

**Methods:**

We performed a retrospective institutional data comparison of percutaneous (PCP Group, *n* = 138) and perventricular (PVP Group, *n* = 67) ultrasound-guided device closure procedures in 205 patients with PmVSD between March 2017 and December 2022.

**Results:**

Patients of the PCP and PVP groups had a median age of 4.9 years (IQR, 3.1–14.0) and 5.3 years (IQR, 3.4–13.1) respectively. The median PmVSD diameter in the PCP Group was 4.0 mm (IQR, 3.3–5.3) and 5.2 mm (IQR, 4.0–7.0) in the PVP Group (*p* = 0.001). There was no significant difference in success rates between the PCP and PVP Groups (intention-to-treat population, 88.4% vs. 92.5%, *p* = 0.36; as-treated population, 88.4% vs. 89.3%, *p* = 0.84). 5/8 failed percutaneous cases that were shifted to the perventricular approach were successful. Compared to the PVP Group, patients of the PCP group experienced a significant decrease in ventilation time, drainage volume, and postoperative hospital stay (*p* < 0.001). The median follow-up period was 24 months (IQR, 6–42) for the PCP group and 61 months (IQR, 53–65) for the PVP group. The overall severe adverse event rate was 0% in the PCP group and 3.0% in the PVP group.

**Conclusions:**

Perventricular and percutaneous ultrasound-guided device closure of PmVSD are both effective and safe treatment options. The percutaneous approach offers less trauma and faster recovery and may be the preferred approach in selected patients.

## Introduction

Perimembranous ventricular septal defects (PmVSD) represent 80% of ventricular septal defects (VSDs) (1) and are closed using percutaneous transcatheter devices with good medium-term outcomes (2, 3). Over the past decade, Chinese surgeons have proposed a perventricular ultrasound-guided device closure to avoid latent radiation-associated hazards (4–8). However, this procedure is associated with surgical morbidity and scarring. To avoid that, Wang et al. performed ultrasound-guided percutaneous PmVSD closure and reported the safety and feasibility of this approach with satisfactory results (9).

Despite these encouraging findings, studies on ultrasound-guided percutaneous PmVSD closures are still rare. Moreover, no previous studies have compared the outcomes between perventricular and percutaneous approaches for ultrasound-guided PmVSD closure. Therefore, we aimed to compare our institutional experience with perventricular and percutaneous ultrasound-guided device closure of PmVSD and describe the outcomes of these interventions.

## Patients and methods

### Study design

We retrospectively reviewed the clinical data of patients who underwent transcatheter percutaneous and perventricular ultrasound-guided PmVSD closure at our institution between March 2017 and December 2022. We divided the patients into percutaneous (PCP) and perventricular (PVP) procedure groups according to the applied closure approach. Standard safety and midterm outcomes were compared. All procedures contributing to this work comply with the ethical standards of the relevant national guidelines on human experimentation, and with the Helsinki Declaration of 1975, as revised in 2008. Approval from the institutional review board was obtained. Written informed consent was signed by the patients or their legal guardians to perform the procedure and to use their clinical records for eventual publication. We extracted in-hospital data from the institutional information system. Echocardiography and electrocardiography reports completed by other health organizations were collected.

### Patient selection and pre-procedure ultrasound evaluation

Patients with congenital PmVSD who met the following criteria were sent for ultrasound-guided device closure: (1) age ≥ 6 months, (2) PmVSD diameter > 2 mm on ultrasound, and (3) absence of cardiovascular malformations requiring surgical repair. Patients with (1) severe pulmonary hypertension, (2) significant aortic valve prolapse without a deep aneurysm or aortic regurgitation ≥ Grade 1, and (3) active infective endocarditis were excluded.

### Echocardiographic assessment

A comprehensive transthoracic echocardiography (TTE) assessment was performed preoperatively by an experienced echocardiologist (J. W.) using a Philips iE33 ultrasound machine (S5-1 probe, 1–5 MHz; S8-3 probe, 3–8 MHz; Philips Medical Systems, Cleveland, Ohio, USA). The GE Vivid E95 (M5SC-D probe, 1.5–4.6 MHz; 6VT probe, 3.0–8.0 MHz; 9 T probe, 4.0–10.0 MHz; GE Healthcare, Chicago, Illinois, USA) was used for intraoperative guidance. We measured the left ventricular entry and right ventricular exit diameters of the pmVSD and focused on the exit diameter as a reference for selecting the occluder size. Residual shunts were classified according to the width of the colored jet. Tricuspid and aortic regurgitations were evaluated using a color-flow Doppler signal and classified into four grades: none or trivial (0/4), mild (1/4), moderate (2/4), and severe (3/4) (10, 11).

### Device and delivery system

The HeartR™ VSD occluder (Lifetech Scientific Corporation, Shenzhen, China), had been described in detail previously (12). It is a self-expandable double-disk device with a 3 mm long connecting waist. The waist diameter correspond to the size of the VSD occluder. Three types of occluders were used: symmetrically concentric, asymmetrically concentric, and eccentric (Supplementary Figure S1). In the symmetrically concentric type, the flanges of both disks are 2 mm wide. In the eccentric type, the aortic flange of the left disk is 0.5 mm wide, whereas its opposite flange is 5 mm wider than the waist. In the asymmetrically concentric type, which is used for PmVSDs with multiple exits, the flange of the left disk is 4 mm and that of the right disk is 2 mm wide. The device selection was based on the protocol that had been previously described (12). An occluder 1–2 mm larger than the targeted shunt of the PmVSD was chosen.

A 20 cm long delivery system with a trocar, a 0.035 inch guidewire, dilator, and loading sheath were chosen for perventricular PmVSD closure. A 90 cm long delivery system was selected for percutaneous PmVSD closure.

### Procedures

All procedures were performed by a hybrid team of cardiac surgeons and echocardiographers under general anesthesia, heparinization, and antibiotic prophylaxis in a routine operating room. Since 2017, we've used perventricular closure for eligible patients. From 2018, with increased percutaneous experience, we've employed the transfemoral retrograde approach for PmVSD closure if the femoral artery diameter is sufficient and the aortic valve interference risk is low (PmVSD with a deep aneurysm or sufficient subaortic rim). For cases where the symmetrical occluder might interfere with the aortic valve, we attempted closure using an eccentric occluder via the femoral vein. If unsuccessful, we resort to perventricular closure.

### Perventricular device closure

Transesophageal echocardiography (TEE) was used to guide the procedure. A 3 cm incision was made in the inferior median sternum. After the sternum was split and retracted, the pericardium was incised and suspended. The optimal puncture site was identified using TEE. After systemic heparinization (1 mg/kg), the rest of the procedure was performed using a previously described protocol (5, 6).

### Percutaneous device closure

**Figure 1:**
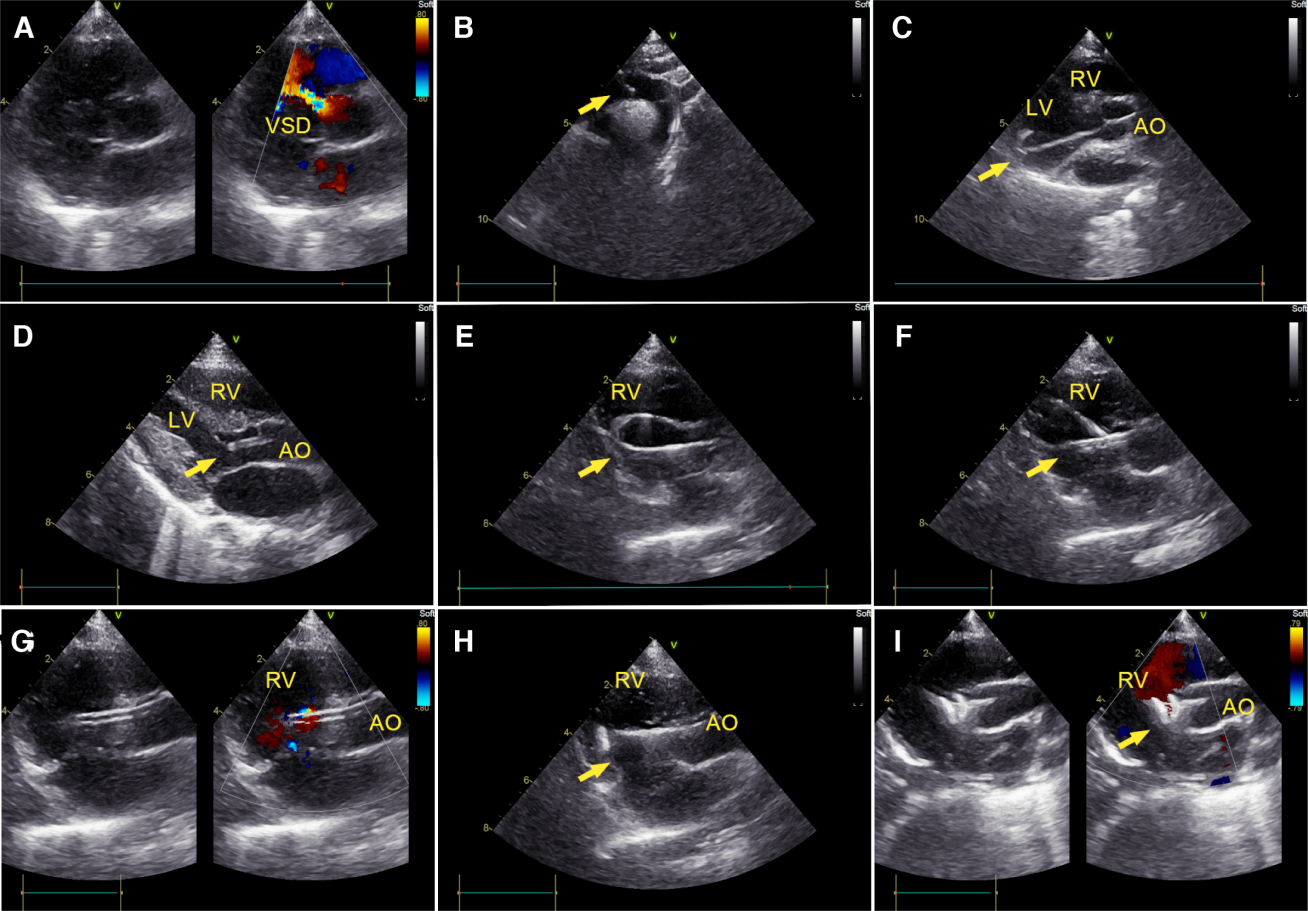
Percutaneous ultrasound-guided perimembranous ventricular septal defect (PmVSD) closure. (**A**) Image of PmVSD in the short- axis view. (**B**) A 5-French trimmed pigtail catheter was introduced along a 0.035-inch angled hydrophilic guidewire (Radifocus Guidewire M, Terumo Medical Corporation, Tokyo, Japan) into the descending aorta, then advanced across the aortic arch in the suprasternal aortic arch long-axis view of TTE. (**C**) Using the long-axis view of the LV, the guidewire crossed the aortic valve, then the pigtail catheter would be advanced into the left ventricle (LV). (**D**) The cut-pigtail catheter was pulled to the level of the PmVSD, and its trimmed tip was rotated toward the ventricular septum in the LV long axis or the apical five-chamber view. (**E**) After the pigtail catheter was advanced to the right ventricle (RV) along the hydrophilic guidewire, a 260 cm exchange J-tip guidewire (Cordis Corporation, Miami Lakes, Florida, USA) was advanced along the catheter into the RV. Its soft tip (arrow) was confirmed in the parasternal long axis view of the RV inflow tract. (**F**) The long delivery sheath (arrow) was inserted over the exchange J-tip guidewire into the RV. (**G**) The guidewire was pulled back and the “double track sign” was viewed. The tip of sheath was confirmed in the RV. (**H**) The occluder was placed inside the delivery sheath, and its right disc (arrow) was deployed. (**I**) The PmVSD was closed after both discs of device were deployed. LV, left ventricle; RV, right ventricle; AO, ascending aorta.

Figure 1 shows the steps of closure under TTE guidance. Before the occluder was unscrewed, the correct position of the device, presence of a residual shunting, and valve regurgitation were identified using multiple echocardiographic views. TEE was performed when TTE images were unclear during the procedure.

When an asymmetrically concentric or eccentric occluder was used, an antegrade approach via the femoral vein was performed according to the method described by Bu et al. (13). We defined device time as the duration from guidewire entry of the guiding sheath to delivery sheath retrieval.

### Postoperative management and follow-up

A daily dose of 3–5 mg/kg oral aspirin was administered after the procedure and continued for six consecutive months. All patients underwent electrocardiogram and TTE before discharge and at 1, 3, 6, and 12 months postoperatively and yearly thereafter.

### Adverse event assessments

We defined major adverse events as all-cause death, cardiovascular perforation, device embolization, large residual shunting at last follow up, new-onset of ≥Grade 2 valvular regurgitation, new-onset of grade II or III atrioventricular block, new-onset complete left bundle branch block, new-onset junctional rhythm, and device-related endocarditis. We defined minor adverse events as small or moderate residual shunting at last follow up, device-related grade 1 valvular regurgitation, pericardial effusion, new-onset left anterior fascicular block, new-onset right bundle branch block, thromboembolism, hemolysis, delayed wound healing, and groin hematoma.

### Statistical analysis

Statistical analyses were performed using R software version 4.2.2. Categorical variables were reported as frequency and percentage and continuous variables were represented as median with interquartile range (IQR). Statistical analyses for continuous variables were conducted using Mann–Whitney U and by chi-square test for categorical variables. Missing data were addressed using the chained equations method for multiple imputations (14, 15). Regression analyses, both linear and logistic, were employed to determine the relationship between closure approaches and various outcomes, after adjusting for confounding factors in two separate models. Sensitivity analyses were conducted on the complete dataset without imputations. A *p*-value < 0.05 was considered statistically significant. All reported *p* values are two-sided.

## Results

### Patient characteristics

We studied 205 consecutive patients (55.6% women/girls) with a median age of 5.0 years (IQR: 3.4–13.8). Among them, 138 (67.3%) had percutaneous procedures, and 67 (32.7%) had perventricular procedures. The median PmVSD diameter in the PCP group was 4.0 mm (IQR, 3.3–5.3) and 5.2 mm (IQR, 4.0–7.0) in the PVP group (*p* = 0.001). Multihole PmVSDs were more common in the perventricular group (28.4% vs. 16.7%, *p* = 0.03) (Table 1).

**Table 1:** Baseline characteristics of the patients.

Variable	Total (*n* = 205)	PCP Group (*n* = 138)	PVP Group (*n* = 67)	*p* Value
Age, year	5.0 (3.4,13.8)	4.9 (3.1,14.0)	5.3 (3.4,13.1)	0.73
Age groups				0.97
≤3 year	53 (25.9)	35 (25.4)	18 (26.9)	
4–14 year	103 (50.2)	70 (50.7)	33 (49.3)	
≥14 year	49 (23.9)	33 (23.9)	16 (23.9)	
Gender				0.20
Male	91 (44.4)	57 (41.3)	34 (50.7)	
Femal	114 (55.6)	81 (58.7)	33 (49.3)	
Height, cm	109.0 (96.0, 148.8)	109.5 (97.0, 149.8)	108.0 (92.5, 147.0)	0.76
Weight, kg	18.0 (14.0, 42.0)	18.0 (14.0, 41.2)	18.0 (13.2, 45.0)	0.47
BSA, m^2^	0.7 (0.6, 1.4)	0.7 (0.6, 1.4)	0.7 (0.6, 1.3)	0.78
Echocardiography				
VSD size, mm	4.5 (3.5, 6.0)	4.0 (3.3, 5.3)	5.2 (4.0, 7.0)	0.001
Missing value	1 (0.5)	0 (0.0)	1 (1.5)	
Subaortic rim < 1 mm	64 (31.2)	38 (27.5)	26 (38.8)	0.06
Missing value	1 (0.5)	0 (0.0)	1 (1.5)	
Multi-hole VSD	42 (20.5)	23 (16.7)	19 (28.4)	0.03
Missing value	1 (0.5)	0 (0.0)	1 (1.5)	
Combined with other malformation	48 (23.4)	40 (29.0)	8 (11.9)	0.004
Missing value	1 (0.5)	0 (0.0)	1 (1.5)	
Shape of VSD				0.02
Window-like	4 (2.0)	3 (2.2)	1 (1.5)	
Infundibular	59 (28.8)	33 (23.9)	26 (38.8)	
Aneurysmal	116 (56.6)	82 (59.4)	34 (50.7)	
Tubular	24 (11.7)	20 (14.5)	4 (6.0)	
Missing value	2 (1.0)	0 (0.0)	2 (3.0)	
TR severity				0.02
0 = none/trace	190 (92.7)	125 (90.6)	65 (97.0)	
1 = mild	14 (6.8)	13 (9.4)	1 (1.5)	
Missing value	1 (0.5)	0 (0.0)	1 (1.5)	
AR severity				NA
0 = none/trace	204 (99.5)	138 (100.0)	66 (98.5)	
1 = mild	1 (0.5)	0 (0.0)	1 (1.5)	
PH				0.95
None	175 (85.4)	117 (84.8)	58 (86.6)	
Mild	21 (10.2)	15 (10.9)	6 (9.0)	
Moderate	7 (3.4)	5 (3.6)	2 (3.0)	
Missing value	2 (1.0)	1 (0.7)	1 (1.5)	
LVEF, %	70.0 (67.0, 74.0)	71.0 (67.8, 74.0)	69.5 (65.0, 74.0)	0.22
Missing value	1 (0.5)	0 (0.0)	1 (1.5)	
Electrocardiography				
cRBBB	4 (2.0)	2 (1.4)	2 (3.0)	0.41
iRBBB	17 (8.3)	10 (7.2)	7 (10.4)	0.42
LAFB	1 (0.5)	1 (0.7)	0 (0.0)	0.60
PVC	3 (1.5)	1 (0.7)	2 (3.0)	0.22

Data are presented as median (IQR) or *n* (%), unless otherwise specified.

IQR, interquartile range; BSA, body surface area; TR, tricuspid valve regurgitation; AR, aortic valve regurgitation; PH, pulmonary hypertension; LVEF, left ventricular ejection fraction; cRBBB, complete right bundle branch block; iRBBB, incomplete right bundle branch block; LAFB, left anterior fascicular block; PVC, premature ventricular contraction.

### Intraoperative and postoperative results

**Figure 2:**
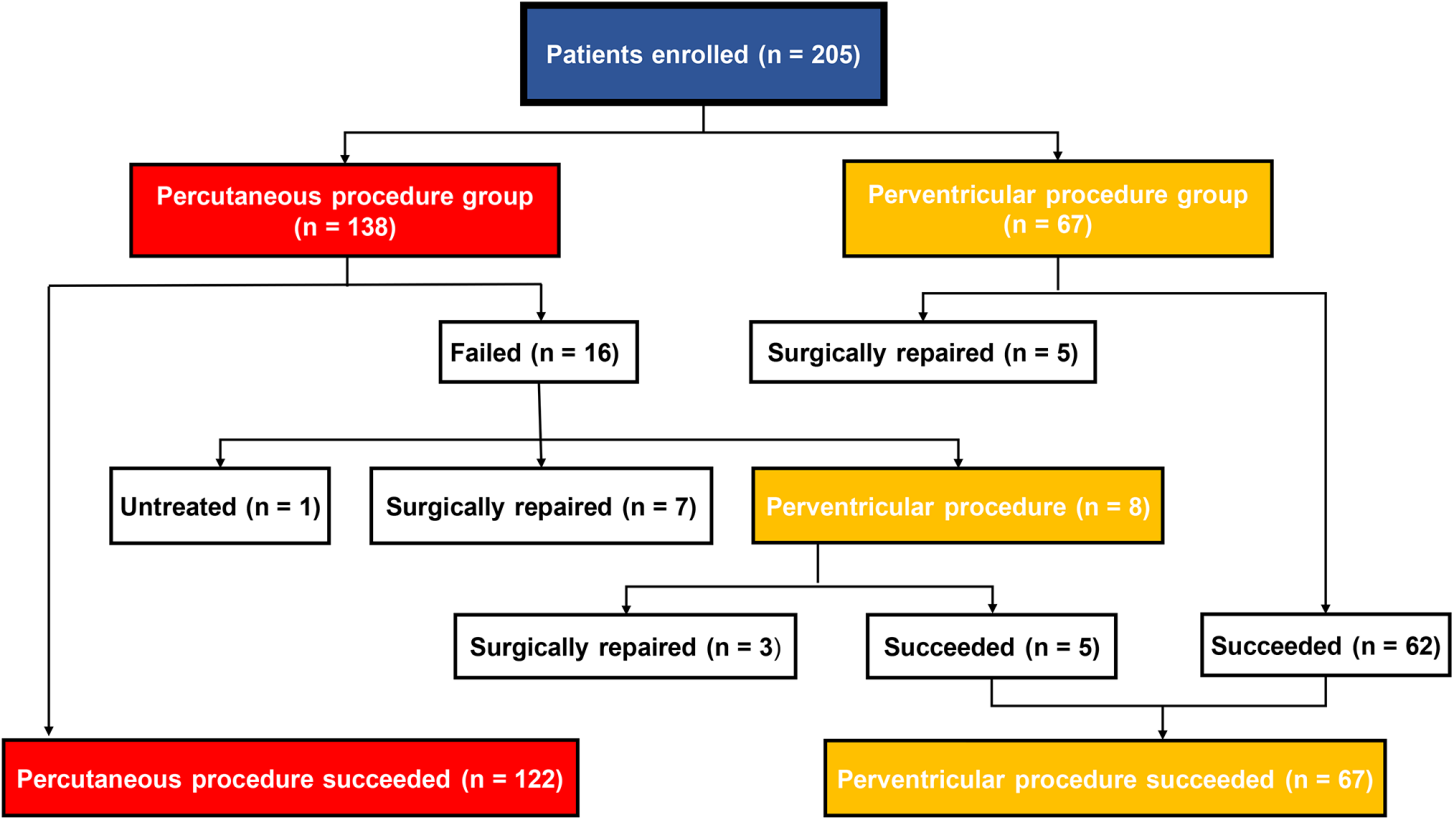
Flow chart of patient inclusion.

The patient’s flow pattern throughout the study period is illustrated in Figure 2. In the intention to treat the population, the success rates were not significantly different between the two groups, with a success rate of 88.4% (122/138) in the PCP group and 92.5% (62/67) in the PVP group (*p* = 0.36). In the as-treated population, the success rates were 88.4% (122/138) in the PCP group and 89.3% (67/75) in the PVP group (*p* = 0.84). In the PCP group, the procedure was completed via antegrade femoral access in 14.8% (18/122) of the patients. Among the 16 patients in the PCP group who experienced failure during the percutaneous procedure, 8 were subsequently converted to a perventricular approach due to difficulties in track establishment. However, out of these 8 patients, we were unable to successfully establish a track in three. In contrast, no patients in the PVP group were converted to a percutaneous procedure.

The unadjusted assessment of the patients in the PCP group vs. PVP group showed a longer device time (56 min vs. 37 min, *p* < 0.001), shorter ventilation time, less drainage volume, and shorter postoperative length of stay (all *p* < 0.001). While 100% of the cases involving the perventricular procedure were performed under TEE guidance, TTE guidance alone was employed in 42.6% of the percutaneous procedures (*p* < 0.001) (Table 2).

**Table 2:** Procedural results.

Variable	Total (*n* = 205)	PCP Group	PVP Group	*p* Value
Success rate,%	92.2	88.4	92.5	0.36
Reasons for procedure failure				0.36
Track establishment	12 (5.9)	11 (8.0)	1 (1.5)	
New-onset AR ≥ Grade 1	4 (2.0)	3 (2.2)	1 (1.5)	
Large residual shunting	4 (2.0)	1 (0.7)	3 (4.5)	
Large VSD	1 (0.5)	1 (0.7)	0 (0.0)	
Device time, min	50.5 (36.0, 70.0)	56.0 (40.0, 73.0)	37.0 (20.0, 60.0)	<0.001
Types of device				<0.001
Symmetric	167 (88.4)	119 (97.5)	48 (71.6)	
Eccentric	12 (6.3)	1 (0.8)	11 (16.4)	
Asymmetric	10 (5.3)	2 (1.6)	8 (11.9)	
Size of device	5.0 (5.0, 7.0)	5.0 (5.0, 7.0)	6.0 (5.0, 7.0)	0.05
Echocardiography				<0.001
TEE	127 (67.2)	60 (49.2)	67 (100.0)	
TTE	52 (27.5)	52 (42.6)	0 (0.0)	
TTE + TEE	10 (5.3)	10 (8.2)	0 (0.0)	
Residual shunting				0.06
None	173 (91.5)	111 (91.0)	62 (92.5)	
Trivial	4 (2.1)	2 (1.6)	2 (3.0)	
Small	10 (5.3)	9 (7.4)	1 (1.5)	
Moderate	2 (1.1)	0 (0.0)	2 (3.0)	
Ventilation time, min	140.0 (98.0, 240.0)	120.0 (85.0, 174.5)	240.0 (142.5, 316.5)	<0.001
Drainage Volume, ml	0.0 (0.0, 50.0)	0.0 (0.0, 0.0)	80.0 (50.0, 150.0)	<0.001
Post-operative length of stay, day	4.0 (3.0, 5.0)	3.0 (2.2, 3.0)	5.0 (4.0, 6.0)	<0.001

Data are presented as median (IQR) or *n* (%), unless otherwise specified.

AR, aortic regurgitation; TEE, transesophageal echocardiography; TTE, transthoracic echocardiography.

After multivariable adjustment (models 1 and 2), the outcomes were consistent with those of the unadjusted population (Supplementary Table S1). We also performed a sensitivity analysis of 180 patients without missing values, and the results were consistent with the primary findings (Supplementary Table S2).

### Adverse events and follow-up data

For the PCP Group, the median follow-up was 24 months (IQR: 6–42 months), while for the PVP Group, it was 61 months (IQR: 53–65 months). Adverse events during procedure and follow-up are listed in Table 3. The severe adverse event rate was 0% in the PCP Group and 3.0% in the PVP Group. Two major adverse events were observed in the PVP group. In one patient, an ascending aortic perforation caused by the short sheath was found immediately after successful pmVSD closure. The incision was extended, and a full sternotomy was performed. The injured ascending aorta was repaired without removal of the occluder. One patient developed acute endocarditis during the fourth month of follow-up. The patient had aortic valve perforation and new-onset moderate aortic valve regurgitation. He underwent replacement of the aortic valve and removal of the occluder. No occluder impingement was observed on the aortic valve during surgery.

**Table 3:** Adverse events during procedure and follow-up.

Adverse Event	PCP Group (*n* = 122) No. (%)	PVP Group (*n* = 67) No. (%)	*p* Value
Severe adverse events			
All-cause death	0	0	NA
Cardiovascular perforation	0	1 (1.5)	0.35
Device embolization	0	0	NA
Large residual shunting at last FU	0	0	NA
New-onset valvular regurgitation ≥ Grade 2	0	0	NA
New onset II° or III° AVB	0	0	NA
New onset CLLLB	0	0	NA
New onset junctional rhythm	0	0	NA
Endocarditis	0	1 (1.5)	0.35
Minor adverse events			
Small or moderate residual shunting at last FU	2 (1.6)	4 (6.0)	0.19
New-onset valvular regurgitation = Grade 1	5 (4.1)	4 (6.0)	0.72
Pericardial effusion	0	0	NA
New-onset LAFB	1 (0.8)	0	>0.99
New onset RBBB	3 (2.5)	4 (6.0)	0.25
Thromboembolism	0	0	NA
Hemolysis	0	0	NA
Delayed healing of wound	0	0	NA
Hematoma of the groin	0	0	NA
Cumulative rate of adverse events	11 (9.0)	14 (20.9)	0.02

FU, follow up; AVB, atrioventricular block; CLLLB, complete left bundle branch block; LAFB, left anterior fascicular block; RBBB, right bundle branch block.

No severe arrythmia events (new-onset II° or III° atrioventricular block, complete left bundle branch block, or junctional rhythm) occurred in either group. One of the five patients with new-onset Grade 1 valvular regurgitation had mild aortic valve regurgitation. When analyzed separately, the rates of adverse events did not differ significantly between the two groups. However, when taken together, the overall rate of adverse events differed significantly between the PCP and PVP groups (*p* = 0.02).

## Discussion

In this study, we compared two approaches of ultrasound-guided PmVSD closure. Our results showed that percutaneous ultrasound-guided closure has comparable feasibility, safety, and event-free survival outcomes to the perventricular approach. Patients who underwent percutaneous ultrasound-guided procedures experienced reduced surgical trauma, faster procedures, and quicker recovery.

Hijazi et al. first reported transcatheter PmVSD closure using an Amplatzer PmVSD occluder in 2002; this procedure has since become an alternative to traditional surgery (16). Transcatheter PmVSD closure has shown proven medium-term follow-up outcomes in a large number of patients who underwent the procedure (2, 3, 17–19). Recently, perventricular PmVSD closure through a minimal incision under TEE guidance has been performed in China (4, 5). This hybrid procedure offers advantages such as avoidance of cardiopulmonary bypass and potential radiation damage, less surgical trauma, and no limitation in patient weight and peripheral vessels. However, existing surgical injuries can counteract the benefits of fluoroscopy-free guidance. Although Wang and colleagues showed promising clinical results for percutaneous PmVSD closure using TTE alone, the adoption of percutaneous approach was rare (9).

Comparable success rates were found between the two approaches in our study. Since percutaneous and perventricular PmVSD cloure have been standard procedures for over 10 years, the VSD anatomy suitable for closure has been well established (20). We followed these standards in our study. It should be noted that the success rate of percutaneous PmVSD closure in our study were lower than the pooled estimate rate of 97.8% reported in a meta-analysis report (21). This could be attributed to our less experience in operative ultrasound guidance and instrument manipulation in the early phase due to our learning curve with exclusive ultrasound guidance.

### Technical considerations

The greatest challenge in percutaneous PmVSD closure without fluoroscopic guidance is tracking the guidewire and sheath. Unlike fluoroscopy, which can display all interventional instruments on the screen, echocardiographic assessments yield 2-D images; therefore, ultrasonologist experience is an important factor for accurate monitoring of the instruments. We emphasized three aspects of procedural safety: tracking the tips of interventional instruments with multiple echocardiographic sections, guidewire protection when the catheter or sheath was advancing, and safe distance measurements for the sheath. However, blind or uncertain manipulation should be avoided. Finally, TEE was used as a supplementary modality when TTE could not yield clear images. None of the patients had to undergo surgery because of dim ultrasonic images. One child experienced an aortic injury during the perventricular procedure in the early phase of the implementation of this technique. No patient experienced bleeding complications due to accidental instrument injury during the percutaneous procedure.

Perventricular device closure via a minimal lower-sternal incision offers a short path and a maneuverable approach that is easy to accomplish. However, a full sternotomy incision is inevitable if the procedure fails. The use of a right thoracic minimal incision can avoid this limitation but is more suitable for children (7, 8). In addition, a lower health-related quality of life in the early postoperative stage of a minimally invasive procedure indicates greater physiological and psychological traumas (22). The present study demonstrated similar results, with the percutaneous group showing a comparable success rate, shorter ventilation time, and shorter postoperative length of stay. The procedure had no limitations in the peripheral vessels when the patients were older than 2 years of age. Therefore, both children and adults can undergo percutaneous PmVSD device closure without fluoroscopy. In most cases, once the procedure fails, a PmVSD repair is performed using a minimally thoracic incision.

### Complications

The anatomical morphology of a PmVSD determines its success rate and long-term outcomes. A previous study showed that the most common reason for crossing over to surgery was new-onset or worsening aortic regurgitation (23). Although the eccentric occluder was designed for VSDs with an insufficient subaortic rim (<1 mm), the success rate of the procedure was associated with prolapse of the aortic valve and sinus (6, 24). In our study, 27.5% of PCP group patients and 38.8% of PVP group patients had a insufficient subaortic rim. Not all cases used eccentric occluders, as symmetrically concentric VSD occluder could close the infundibular or aneurysmal structure without affecting the aortic valve. Despite previous encouraging results by other operators using mixed guidance, VSD closure was avoided in cases without an aneurysmal structure and when the subaortic rim was less than 1 mm (25).

Serious arrythmia rarely occurs during follow-up. However, a higher incidence of serious postoperative arrhythmia has been reported in patients treated using an eccentric occluder (26). The proposed explanation is that the occurrence of a heart block after closure is due to conduction impairments caused by occluder compression. Kaur and colleagues utilized a mapping system to demonstrate several positional relationships between conduction bundles and the PmVSD (27). Therefore, eccentric and asymmetrically concentric occluders may have a higher rate of arrhythmia postoperatively because of their wider disks. We have always been cautious about utilizing these two types of occluders in PmVSD cloure. This may explain the low rate of postoperative arrhythmias observed in our cohort.

## Limitations

First, this was a single-center retrospective study; therefore, selection bias was inevitable. We could not clarify whether these findings could be extended to low-weight children, since our study did not include children weighing less than 10 kg in the percutaneous group. Second, prospective randomized controlled studies are needed to compare these two procedures in patients with similar PmVSD morphologies. Third, a longer follow-up period was required because of the uncertainty of severe complications, such as a complete atrioventricular block or left bundle branch block.

## Conclusions

Perventricular and percutaneous ultrasound-guided device closure of PmVSD are both effective and safe treatment options. The percutaneous approach offers less trauma, faster recovery and may be the preferred approach in selected patients.

## Data Availability

The original contributions presented in the study are included in the article/**Supplementary Material**, further inquiries can be directed to the corresponding author.
