# Supplementary material for: Comparison of perventricular and percutaneous ultrasound-guided device closure of perimembranous ventricular septal defects
Source: Front Cardiovasc Med. 2023 Nov 6;10:1281860. doi: 10.3389/fcvm.2023.1281860 (PMC10657817; doi:10.3389/fcvm.2023.1281860)
Supplement: Supplementary file 1 [file Table1.docx]

**Supplemental Table 1 Adjusted procedure results using** **multiple linear or logistic regression**

| Variable | Model 1 ^a^ | |  | Model 2 ^b^ | |
| --- | --- | --- | --- | --- | --- |
|  | β / OR (95% CI) | P |  | β / OR (95% CI) | P |
| Device time |  |  |  |  |  |
| PCP | Ref. |  |  | Ref. |  |
| PVP | β = -11.40 (-21.91, -0.90) | 0.035 |  | β = -15.78 (-27.44, -4.12) | 0.009 |
| Size of Device |  |  |  |  |  |
| PCP | Ref. |  |  | Ref. |  |
| PVP | β = 0.52 (-0.04, 1.08) | 0.069 |  | β = -0.28 (-0.67, 0.11) | 0.17 |
| Ventilation Time |  |  |  |  |  |
| PCP | Ref. |  |  | Ref. |  |
| PVP | β = 108.77 (71.61, 145.92) | <0.001 |  | β = 118.76 (78.11, 159.41) | <0.001 |
| Drainage |  |  |  |  |  |
| PCP | Ref. |  |  | Ref. |  |
| PVP | β = 107.66 (91.55, 123.77) | <0.001 |  | β = 99.92 (82.15, 117.70) | <0.001 |
| Post-operative Length of Stay |  |  |  |  |  |
| PCP | Ref. |  |  | Ref. |  |
| PVP | β = 1.97 (1.65, 2.30) | <0.001 |  | β = 1.97 (1.62, 2.33) | <0.001 |
| Residual Shunt ^c^ |  |  |  |  |  |
| PCP | Ref. |  |  | Ref. |  |
| PVP | OR = 0.78 (0.26, 2.35) | 0.654 |  | OR = 0.23 (0.04, 1.20) | 0.08 |
| Types of Device ^d^ |  |  |  |  |  |
| PCP | Ref. |  |  | Ref. |  |
| PVP | OR = 12.77 (4.06, 40.19) | <0.001 |  | OR = 16.87 (3.43, 83.05) | <0.001 |

^a^ Model 1 includes adjustment for age and gender.

^b^ Model 2 includes adjustment for age, gender, height, weight, body surface area, diameter of VSD, subaortic rim≤1mm，multi-hole VSD, cardiac malformation, type of VSD, tricuspid regurgitation, pulmonary hypertension, EF, complete right bundle branch block, incomplete right bundle branch block, Left anterior fascicular block and premature ventricular contraction.

^c^ Residual shunt are converted to binary categorical variables, including yes or no；

^d^ Types of Device are converted to binary categorical variables, including symmetric and other types.

PCP, percutaneous procedure; PVP, perventricular procedure;
