# Supplementary material for: Comparison of perventricular and percutaneous ultrasound-guided device closure of perimembranous ventricular septal defects
Source: Front Cardiovasc Med. 2023 Nov 6;10:1281860. doi: 10.3389/fcvm.2023.1281860 (PMC10657817; doi:10.3389/fcvm.2023.1281860)
Supplement: Supplementary file 2 [file Table2.docx]

**Supplemental Table 2 Adjusted procedure results in sensitivity analysis (n=180)**

| Variable | Model 1 ^a^ | |  | Model 2 ^b^ | |
| --- | --- | --- | --- | --- | --- |
|  | β / OR (95% CI) | P |  | β / OR (95% CI) | P |
| Device time |  |  |  |  |  |
| PCP | Ref. |  |  | Ref. |  |
| PVP | β = -12.09 (-23.12, -1.06) | 0.033 |  | β = -15.85 (-28.21, -3.49) | 0.01 |
| Size of Device |  |  |  |  |  |
| PCP | Ref. |  |  | Ref. |  |
| PVP | β = 0.45 (-0.11, 1.02) | 0.119 |  | β = -0.27 (-0.65, 0.12) | 0.17 |
| Ventilation Time |  |  |  |  |  |
| PCP | Ref. |  |  | Ref. |  |
| PVP | β = 105.49 (66.85, 144.13) | <0.001 |  | β = 117.40 (75.46, 159.34) | <0.001 |
| Drainage |  |  |  |  |  |
| PCP | Ref. |  |  | Ref. |  |
| PVP | β = 105.22 (88.79, 121.64) | <0.001 |  | β = 96.90 (78.93, 114.88) | <0.001 |
| Post-operative Length of Stay |  |  |  |  |  |
| PCP | Ref. |  |  | Ref. |  |
| PVP | β = 1.91 (1.57, 2.24) | <0.001 |  | β = 1.95 (1.59, 2.31) | <0.001 |
| Residual Shunt ^c^ |  |  |  |  |  |
| PCP | Ref. |  |  | Ref. |  |
| PVP | OR = 0.63 (0.19, 2.09) | 0.451 |  | OR = 0.20 (0.04, 1.08) | 0.06 |
| Types of Device ^d^ |  |  |  |  |  |
| PCP | Ref. |  |  | Ref. |  |
| PVP | OR = 11.44 (3.58, 36.58) | <0.001 |  | OR = 13.94 (2.96, 65.71) | <0.001 |

^a^ Model 1 includes adjustment for age and gender.

^b^ Model 2 includes adjustment for age, gender, height, weight, body surface area, diameter of VSD, subaortic rim≤1mm，multi-hole VSD, cardiac malformation, type of VSD, tricuspid regurgitation, pulmonary hypertension, EF, complete right bundle branch block, incomplete right bundle branch block, left anterior fascicular block and premature ventricular contraction.

^c^ Residual shunt are converted to binary categorical variables, including yes or no；

^d^ Types of Device are converted to binary categorical variables, including symmetric and other types.

PCP, percutaneous procedure; PVP, perventricular procedure;
