# Supplementary figures and images for: Comparison of perventricular and percutaneous ultrasound-guided device closure of perimembranous ventricular septal defects
Source: Front Cardiovasc Med. 2023 Nov 6;10:1281860. doi: 10.3389/fcvm.2023.1281860 (PMC10657817; doi:10.3389/fcvm.2023.1281860)

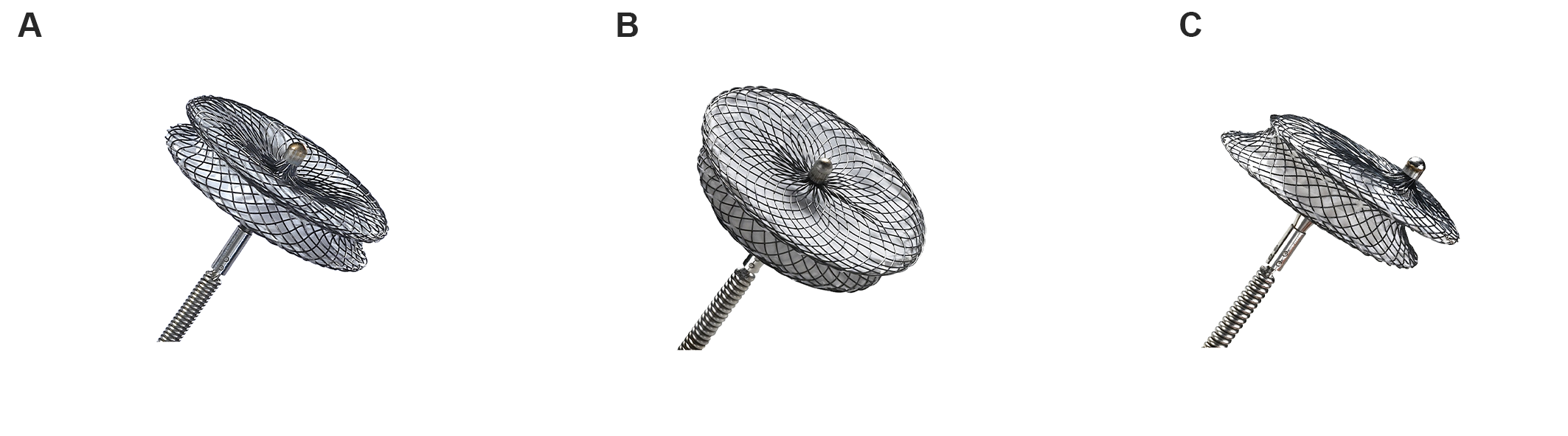

Supplement: Supplementary file 3 [file Image1.tif]
